# High frequency of Polio-like Enterovirus C strains with differential clustering of CVA-13 and EV-C99 subgenotypes in a cohort of Malawian children

**DOI:** 10.1007/s00705-018-3878-7

**Published:** 2018-05-28

**Authors:** Lieke Brouwer, Sabine M. G. van der Sanden, Job C. J. Calis, Andrea H. L. Bruning, Steven Wang, Joanne G. Wildenbeest, Sjoerd P. H. Rebers, Kamija S. Phiri, Brenda M. Westerhuis, Michaël Boele van Hensbroek, Dasja Pajkrt, Katja C. Wolthers

**Affiliations:** 1 Department of Medical Microbiology, Laboratory of Clinical Virology, Academic Medical Center, University of Amsterdam, Meibergdreef 9, 1105 AZ Amsterdam, The Netherlands; 2 Department of Paediatric Intensive Care, Emma Children’s Hospital, Academic Medical Center, Amsterdam, The Netherlands; 3 Department of Paediatric Infectious Diseases, Emma Children’s Hospital, Academic Medical Center, Amsterdam, The Netherlands; 4 School of Public Health and Family Medicine, College of Medicine, University of Malawi, Blantyre, Malawi; 5 Present Address: Department of Paediatrics and Paediatric Infectious Diseases, Wilhelmina Children’s Hospital, University Medical Center Utrecht, Utrecht, The Netherlands; 6 Present Address: Department of Viroscience, Erasmus MC, Rotterdam, The Netherlands

## Abstract

Enteroviruses (EVs) are among the most commonly detected viruses infecting humans worldwide. Although the prevalence of EVs is widely studied, the status of EV prevalence in sub-Saharan Africa remains largely unknown. The objective of our present study was therefore to increase our knowledge on EV circulation in sub-Saharan Africa. We obtained 749 fecal samples from a cross-sectional study conducted on Malawian children aged 6 to 60 months. We tested the samples for the presence of EVs using real time PCR, and typed the positive samples based on partial viral protein 1 (VP1) sequences. A large proportion of the samples was EV positive (89.9%). 12.9% of the typed samples belonged to EV species A (EV-A), 48.6% to species B (EV-B) and 38.5% to species C (EV-C). More than half of the EV-C strains (53%) belonged to subgroup C containing, among others, Poliovirus (PV) 1-3. The serotype most frequently isolated in our study was CVA-13, followed by EV-C99. The strains of CVA-13 showed a vast genetic diversity, possibly representing a new cluster, ‘F’. The majority of the EV-C99 strains grouped together as cluster B. In conclusion, this study showed a vast circulation of EVs among Malawian children, with an EV prevalence of 89.9%. Identification of prevalences for species EV-C comparable to our study (38.5%) have only previously been reported in sub-Saharan Africa, and EV-C is rarely found outside of this region. The data found in this study are an important contribution to our current knowledge of EV epidemiology within sub-Saharan Africa.

## Introduction

Genus *Enterovirus* is a member of the family of *Picornaviridae* and consists of 13 species of which 7 classify as viruses which infect humans, i.e. *Enterovirus A-D* and *Rhinovirus A-C* [1]. Enteroviruses (EV’s) are known to cause a wide variety of clinical symptoms, ranging from mild respiratory infections to invasive disease such as meningitis, encephalitis and acute flaccid paralysis (AFP). Systematic surveillance programs have led to an increased knowledge on EV circulation in large parts of the world. In Europe and the United States, EVs are found in 5-12% of clinical samples [2–6]. Most of the EV strains found in Europe and the USA belong to species B, while species A is dominant in Asia [2, 3, 5, 7–12]. Regular outbreaks of EVs causing severe disease and complications, such as EV-A71 and EV-D68, have been reported in various countries in North-America, Europe and Asia, as well as in Australia [13, 14]. The status of EV prevalence in sub-Saharan Africa remains largely unknown, due to incomplete sampling or data collection. The few data available report a high EV prevalence – up to 50% – with an EV-C proportion of up to 76% amongst circulating EV strains [15–28].

Poliovirus (PV) is the most well-known EV causing AFP [29, 30]. Global vaccination programs have significantly reduced the incidence of PV infections, with PV now being endemic in only three countries (Afghanistan, Pakistan and Nigeria) [31]. PV belongs to species EV-C, and attenuated PV, as administered in the oral polio vaccine (OPV), can recombine with other strains belonging to EV-C to form vaccine-derived poliovirus (VDPV) [32–35]. Outbreaks of such VDPVs causing polio-like symptoms have been reported in the Philippines, Madagascar, the Dominican Republic, Haiti, Cambodia, Nigeria and Egypt [32, 36–40]. A high prevalence of EV-C in sub-Saharan Africa could increase the chances of VDPV’s arising in this continent.

The aim of this study was to provide further insights into the prevalence of EVs in children in sub-Saharan Africa, to assess the distribution of species EV-A, -B and –C, and finally to examine the genetic variability within the circulating species and genotypes. For this, we used fecal samples obtained in a case-control study conducted on children in Malawi. We report a high frequency of EVs classifiable as subgroup C of species C, a group that also contains PV.

## Materials and methods

### Patients and samples

A total of 749 fecal samples obtained from children included in the case-control SevAna (Severe Anemia) study in Southern Malawi between 2002 and 2004 were included in this study. The SevAna study was ethically approved and has been described in detail previously [41]. The samples used in our analyses were obtained from patients with: severe anemia (hemoglobin < 5 g/dl), hospital controls without severe anemia and randomly selected community controls. All included participants were between 6 and 60 months of age. A questionnaire, including date of birth, sex, date of recruitment and discharge and clinical symptoms, was completed for each participant. Fecal samples were stored at -20 °C and shipped to Leiden University Medical Center, the Netherlands. After storage for 10 years, the remaining samples were brought to the Academic Medical Center, Amsterdam for continued storage at -20 °C.

### Virus isolation and detection

The Boom nucleic acid extraction method was used to isolate RNA from each sample [42]. RT-PCR was performed as described previously using primers EV-1 and EV-2 to determine presence of EV in the samples (Table 1) [43]. Samples with a Ct-value < 40 were considered to be EV positive. Samples with a Ct-value < 30 were included for sequencing.

**Table 1:** Primers and probes used for RT-PCR (primers EV-1, EV-2 and probe WT-MGB), semi-nested PCR (primers 224, 222, AN89 and AN88) and sequencing (primers AN89 and AN88)

Primer/probe	Sequence 5’-3’	Polarity	Gene	Genomic location
224	GCIATGYTIGGIACICAYRT	Forward	VP1	1977-1996
222	CICCIGGIGGIAYRWACAT	Reverse	VP1	2969-2951
AN89	CCAGCACTGACAGCAGYNGARAYNGG	Forward	VP1	2602-2627
AN88	TACTGGACCACCTGGNGGNAYRWACAT	Reverse	VP1	2977-2951
EV-1	GGCCCTGAATGCGGCTAAT	Forward	5’UTR	450-468
EV-2	GGGATTGTCACCATAAGCAGCC	Reverse	5’UTR	600-579
WT-MGB (probe)	CGGAACCGACTACTTTGGGT		5’UTR	532-551

### Enterovirus typing and phylogenetic analysis

A sensitive, semi-nested PCR amplification of VP1 sequences was performed, including primers 224 and 222 for the first PCR and primers AN89 and AN88 for the second PCR, as described previously (Table 1) [44]. The size of the PCR fragments was analyzed by gel electrophoresis. Positive samples with a PCR fragment size of ~ 350 to 400 base pairs (bp) were selected and sequenced using the BigDye Terminator kit, together with primers AN89 and AN88. CodonCode Aligner was used to assemble the obtained sequences. The sequences were typed using the online RIVM enterovirus genotyping tool (National Institute for Public Health and the Environment, http://www.rivm.nl/mpf/typingtool/enterovirus/ accessed 1st December 2014) and by comparison with reference strains in GenBank using BLAST (NCBI, https://blast.ncbi.nlm.nih.gov/ accessed 1st December 2014). The VP1 sequences obtained in this study were aligned with GenBank reference strains for respective genotypes, using ClustalX2 software. Neighbor-joining trees were constructed of study strains and reference strains, using the p-distance model implemented in MEGA 6 Software. One thousand bootstrap replicates were used to test the support for branches within the tree.

The nucleotide sequence data reported in this paper will appear in the DDBJ/EMBL/GenBank nucleotide sequence databases with the accession numbers MG793383-MG793425.

### Statistical analysis

Baseline characteristics were calculated as frequencies and percentages for categorical variables, and as median and interquartile range (IQR) for numerical variables. We examined associations between EV positivity and the variables sex, inclusion group and age by Fisher’s exact test and Mann-Whitney-U test. Within the community control group, we examined the association between possible EV-related symptoms (i.e. gastro-intestinal symptoms, respiratory symptoms, central nervous system symptoms and fever) and EV infection using logistic regression analysis, and corrected for sex and age. Since 38.2% of the community control group was diagnosed with malaria, we also corrected for diagnosis of malaria. All statistical analyses were performed using IBM SPSS Statistics 24. Correlations were considered to be significant at an alfa-level of 0.05 or lower.

## Results

### EV prevalence in Malawian children

The baseline characteristics of the study participants are shown in Table 2. Baseline characteristics, sex and median age were comparable among the three inclusion groups.

**Table 2:** Baseline characteristics

	Cases with severe anemia, n = 227	Hospital controls, n = 261	Community controls, n = 249	Total^a^ (n = 749)
Patient characteristics				
Male sex, n (%)	107 (47)	133 (51)	120 (48)	371 (50)
Age in years, median (IQR)	1.30 (0.85-2.16)	1.76 (1.06-2.39)	2.00 (1.20-3.01)	1.64 (1.02-2.60)
EV-positive, n (%)	198 (87)	234 (90)	229 (92)	673 (90)
Sequenced, n (%)^b^	77/198 (39%)	104/234 (44%)	99/229 (43%)	283/673 (42%)
EV-A, n (%)	15 (19)	8 (8)	15 (15)	38 (13)
EV-B, n (%)	37 (48)	58 (56)	40 (40)	137(48)
EV-C, n (%)	25 (32)	38 (37)	44 (44)	108 (38)

**Fig. 1:**
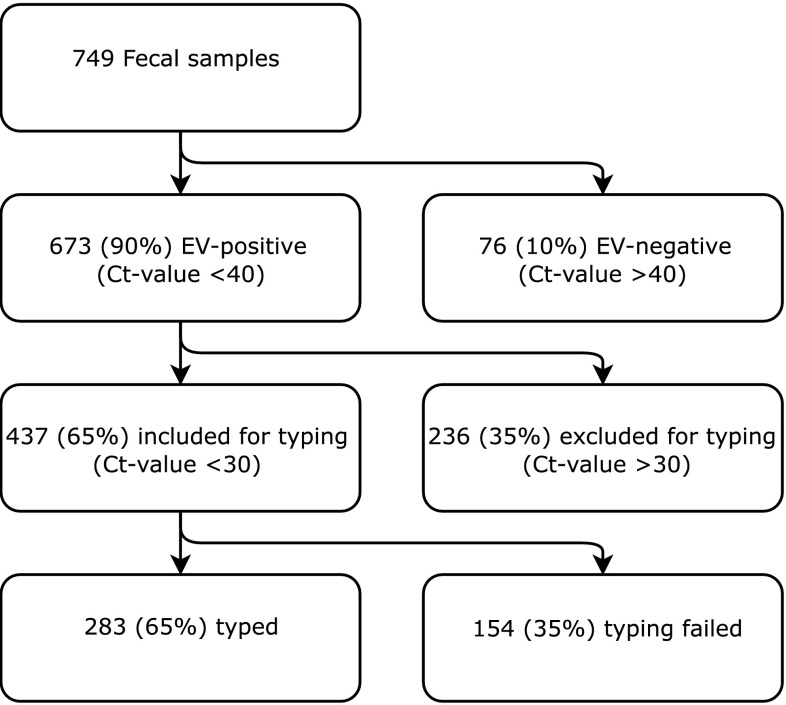
Flowchart for all the included samples

Overall, 673 of the 749 fecal samples (89.9%) tested positive for EV targeting the 5’UTR by qPCR. Of 437 samples included for genotyping, good quality sequences could be retrieved from 283/437 (65%) of samples (Figure 1). In total, we found 59 different genotypes (Table 3). Enterovirus B was the most frequently detected species, followed by Enterovirus C and Enterovirus A (53%, 34% and 13%, respectively) (Table 3). No Enterovirus D was detected.

**Table 3:** List of all typed strains

	No. of typed strains	% of all typed strains
**Species and type**		
**HEV-A**		
CVA-2	2	0.7%
CVA-4	2	0.7%
CVA-5	4	1.4%
CVA-6	5	1.7%
CVA-7	1	0.3%
CVA-8	2	0.7%
CVA-10	2	0.7%
CVA-14	1	0.3%
CVA-16	2	0.7%
EV-A76	3	1.0%
EV-A89	2	0.7%
EV-A119	8	2.8%
EV-A120	3	1.0%
*All HEV-A*	*37*	*12.9%*
**HEV-B**		
CVA-9	9	3,1%
CVB-2	1	0.3%
CVB-4	1	0.3%
CVB-5	1	0.3%
E1	7	2.4%
E2	2	0.7%
E5	5	1.7%
E6	10	3.5%
E7	4	1.4%
E9	1	0.3%
E11	4	1.4%
E12	1	0.3%
E13	7	2.4%
E14	9	3.1%
E15	12	4.2%
E18	6	2.1%
E19	5	1.7%
E20	3	1.0%
E21	4	1.4%
E24	1	0.3%
E25	3	1.0%
	5	1.7%
E29	4	1.4%
E33	4	1.4%
EV-B69	1	0.3%
EV-B73	1	0.3%
EV-B75	5	1.7%
EV-B78	4	1.4%
EV-B79	2	0.7%
EV-B82	4	1.4%
EV-B80	8	2.8%
EV-B88	2	0.7%
EV-B97	1	0.3%
EV-B100	2	0.7%
*All HEV-B*	*139*	*48.6%*
**HEV-C**		
CVA-1	9	3,1%
CVA-11	7	2.4%
CVA-13	34	11.9%
CVA-17	3	1.0%
CVA-19	1	0.3%
CVA-20	12	4.2%
CVA-24	8	2.8%
EV-C99	31	10.8%
EV-C116	3	1.0%
PV2 (Sabin-like)	1	0.3%
PV3 (Sabin-like)	1	0.3%
*All HEV-C*	*110*	*38.5%*
**All typed strains**	**286**	**100%**

Within EV-B, the most frequently detected genotypes were echovirus 6 (10/286, 3.5%) and echovirus 15 (12/286, 4.2%), while several of the higher numbered EV-B genotypes were also detected (EV-B69-100). In EV-C, CV-A13 (34/286, 11.9%) and EV-C99 (31/286 10.8%) were the most frequently detected genotypes. EV-A119 (8/286, 2.8%) and CVA6 (5/286, 1.7%) were the genotypes most frequently detected within EV-A (Table 1). One PV2-strain and one PV3-strain were identified, both of which were shown to have a ≥ 98.6% match to their respective reference Sabin strains in GenBank, indicating that these are vaccine-like polioviruses.

Enterovirus C has previously been divided into subgroups A, B and C [45]. Of all the typed EV-C strains in our study, 53% belonged to subgroup C, 35% to subgroup B and 12% to subgroup A.

EV prevalence was not associated with age (p = 0.882), sex (p = 0.629) or study group (p = 0.250). Within the community control group, the reporting of possible EV-related symptoms was not correlated with EV infection (p = 0.531).

### Genetic diversity of EV-C strains

**Fig. 2:**
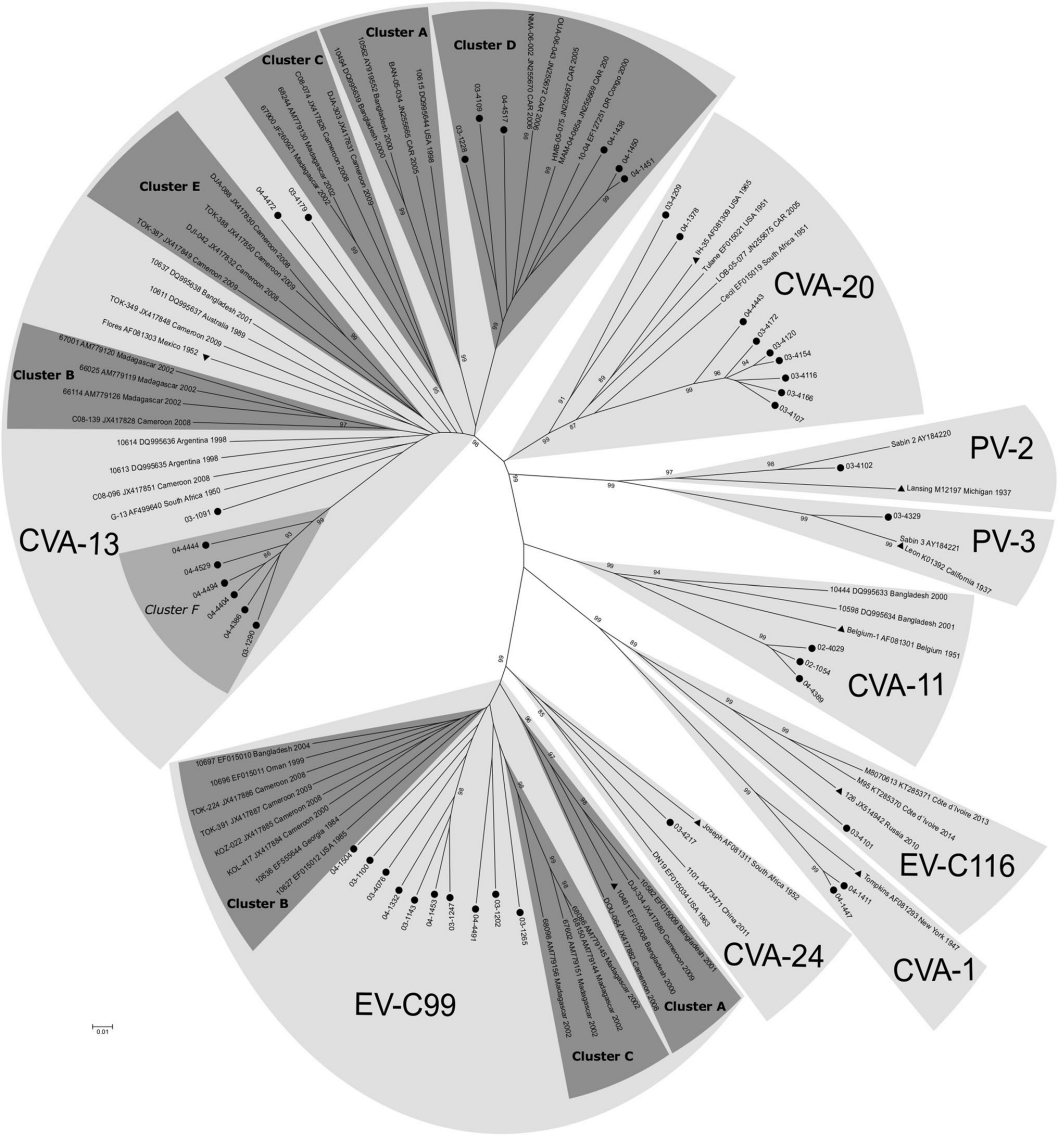
Phylogenetic relationships, based on the VP1 3’ terminal nucleotide sequences, for Malawian field strains and reference strains available in GenBank. Supportive percentage bootstrap replicates ≥ 85% are shown. Studied strains are indicated by circles. For reference strains, the location and year of isolation are indicated (DR Congo, Democratic Republic of the Congo; CAR, Central African Republic). The prototype strains are indicated by triangles. Cluster F is marked in light grey to indicate that this is a potential new cluster, based on the nt and aa percentage similarity to the other clusters

Figure 2 shows the genetic relationship of our Malawian EV-C strains with respective reference strains obtained from GenBank.

All strains grouped with their corresponding reference strains into type-specific clusters with strong bootstrap support (Figure 2). The nucleotide (nt) and amino acid (aa) identity within the serotypes was ≥ 75% and ≥ 88% respectively for all types except for CVA-13. For CVA-13 it was ≥ 70.0% and ≥ 84.3%, respectively.

As reported previously, the CVA-13 sequences grouped into clusters A to D, with ≥ 79.2% and ≥ 91.7% nt and aa identity within each of these clusters (Table 4) [27, 46]. Five of the Malawian CVA-13 sequences clustered with strains from the Central African Republic and the Democratic Republic of the Congo in CVA-13 Cluster D (bootstrap value 100%). The nt and aa identity within this cluster was ≥ 81.5% and ≥ 91.5%, respectively (Table 4). The nt and aa identity of cluster D compared to the other clusters was 69.3%-73.3% and 79.7%-89.2%, respectively, whereas the identity scores between clusters A, B, C and E were 71.3%-77.3% for nt, and 81.2%-94.0% for aa identity (Table 4).

**Table 4:** Nucleotide (Nt) and amino acid (Aa) identity scores; identity scores are given within each CVA-13 cluster (minimum identity scores), and in comparison between CVA-13 clusters

	Nt identity	Aa identity
Within CVA-13 clusters		
*Cluster A*	≥ 80.6%	≥ 93.1%
*Cluster B*	≥ 79.4%	≥ 96.9%
*Cluster C*	≥ 78.5%	≥ 93.7%
*Cluster D*	≥ 81.5%	≥ 91.5%
*Cluster E*	≥ 78.8%	≥ 91.8%
*Cluster F*	≥ 89.1%	≥ 95.3%
Between CVA-13 clusters		
*Between cluster A, B, C and E*	72.3%-77.3%	86.4%-94.0%
*Cluster D compared to cluster A, B, C and E*	69.0%-73.3%	79.7%-89.2%
*Cluster F compared to cluster A, B, C, D and E*	69.0%-77.0%	82.4%-94.6%

(range of minimum through maximum identity scores)

Furthermore, seven Malawian CVA-13 strains, as well as several reference strains, did not belong to a known cluster. Of these, six of our sequences clustered together, supported by a bootstrap value of 99%, suggesting a new cluster, ‘F’ (Figure 2). The nt and aa identity within this cluster was 89.1%% and 95.3% respectively, while the nt and aa identity compared to the other clusters was 69.0%-77.0% and 82.4%-94.6% respectively (Table 4).

EV-C99 is known to consist of three clusters, i.e. A, B and C [27, 46], supported by bootstrap values of 96%, 5% and 97% respectively in our phylogram. Our strains did not group with any of the clusters. Three of our strains (03-1265, 03-1202 and 04-4491) were most similar to cluster C (≥ 80.8% nt identity and ≥ 92.6% aa identity). The remaining seven strains were most closely related to cluster B (≥ 80.8% nt identity and ≥ 94.9% aa identity).

## Discussion

Our study contains new information about EV epidemiology and genetic diversity within sub-Saharan Africa, which contributes to our knowledge on EV-C circulation. Since epidemiological data from sub-Saharan Africa are scarce and the circulation of EV-C is focused upon in light of the PV eradication campaign, data from older cohorts like ours are still highly relevant. We detected EV in 89.9% of fecal samples collected from children between 2002 and 2004 in two hospitals in southern Malawi. This EV frequency is higher than in previous studies from sub-Saharan Africa, that reported EV prevalence numbers ranging from 1.5% to 50% [15–28]. Furthermore, it exceeds the 50% EV prevalence that has previously been reported in Malawi [26]. This difference might be partially explained by several factors. Firstly, in our study, we used real time PCR for detection of EV in fecal samples, whereas until recently, cell culture and virus isolation was the method most often used to detect EVs. 5’UTR PCR has been shown to detect EV from clinical specimens with a higher sensitivity than cell culture, resulting in higher yields especially for the non-B viruses [4, 6, 47]. Secondly, we hypothesize that our high EV prevalence is further explained by our relatively young population. While other studies often focus on a broader age group, EV’s are more prevalent in young age groups, when compared to older children and adults [15, 19]. Thirdly, the inclusion criteria of the SevAna study led to a higher number of participants included during the rainy season, in which malaria, a well-known cause of anemia, is highly prevalent. Possible seasonal variation in EV prevalence, much like in the Western world, might therefore have led to a higher detected prevalence in our study.

Interestingly, while we included one sample for each study participant, high EV incidence numbers have been found in studies that included multiple samples from children followed over a longer time-period. One study in Kenya showed that in a group of HIV-positive children 92% had at least one EV positive fecal sample during a 1-year study period [21]. A study conducted in Norway found that 90% of healthy children had shed EV at least once during a two-year follow-up [48].

In our study, 34% of the typed EV strains belonged to species EV-C. Although EV-C is a rather rare species in most of the world [3, 7, 8, 49–51], it accounts for up to 76% of typed EV strains found in African populations [18, 20, 22, 25–28]. The high proportion of EV-C subgroup C as found in our study is in accordance with findings in Cameroon and Madagascar. Furthermore, the types within EV-C that were most frequently detected in our study (CVA-13, CVA-20, EV-C99 and CVA-24) are found in approximately the same proportions in Cameroon and Madagascar [27, 52].

We saw a vast genetic diversity within EV-C subgroup C, especially within serotype CVA-13. It has been reported by others that CVA-13 strains group together in clusters (A-E) [27, 46]. In our phylogram, we could see this clustering, although cluster B and E were supported by low bootstrap values (73% and 33% respectively). Cluster D seems genetically distinct from the other clusters, with the maximum nt identity percentage compared to the other clusters falling below 75% (73.3%, table 4). Furthermore, several of the CVA-13 strains in our study did not fall within any of the clusters. Four of those strains grouped together, possibly forming a new cluster ‘F’.

For EV-C99, cluster B in our phylogenetic tree is merely supported by a bootstrap value of 5%. This is most likely a result of several of our strains grouping close to cluster B. Even so, the joint group of cluster B and our strains is supported by a bootstrap value of merely 25%.

We found PV in two of our samples (one strain PV-2 and one strain PV-3). Since the oral polio vaccine is administered at birth, this low prevalence is in accordance with our participants being between 6 and 60 months of age. The PV strains found in our study are most likely derived from children who had received a boost dose, or by secondary spread of the vaccine.

We found several strains of recently discovered genotypes EV-A119, EV-A120 and EV-C116. The prototype strains of these genotypes are derived from samples obtained years after the collection date of samples analyzed in our study [53–55]. We found eight strains of EV-A119, whereas the oldest known reference strain dates back to 2008 [52]. EV-A119 has only been detected in three children in Cameroon, Côte d’Ivoire and Nigeria [16, 18, 56]. The large proportion of EV-A119 in our database is therefore remarkable. In contrast, EV-A71, circulating widely in Asia and Europe, and also reported in several studies in sub-Saharan Africa, was not detected in our population [7, 27, 57]. Furthermore, we found several EV-B genotypes – Echo 1, Echo 15 and types EV-B69-100 – that are rarely found in Asia, Europe and the US, but seem to be rather prevalent in sub-Saharan Africa [7, 18, 27, 28, 57].

The major limitation of our study is the sample collection taking place between 2002 and 2004. Over time, the circulation and distribution of genotypes might have changed. However, the high diversity within CVA-13 found in our study and the repeated isolation of this type throughout the whole study period is interesting and suggests continuous circulation. Furthermore, making use of sequence based typing, which was not available at the time of sample collection, gives a unique insight into an older sample set, e.g. revealing circulation of EV-A119 before the first strain was even identified.

In conclusion, we found high rates of EV prevalence in young children in Malawi and high rates of EV-C – specifically of subgroup C within EV-C. High EV-C circulation is worrying, as strains belonging to this species are able to recombine with PV, giving rise to virulent VDPV strains. Furthermore, we saw a vast genetic diversity within CVA-13. Further studies using full length sequences of our study strains should reveal whether and to what scale recombined EV-C strains containing PV fragments are circulating within our population. Moreover, these future studies will also show the exact genetic diversity within CVA-13 – focusing on the genetic variety of cluster D when compared to the other clusters, as well as the genetic diversity of cluster F.

## Abbreviations

EV: Enterovirus
PV: Poliovirus
CV: Coxsackievirus
OPV: Oral polio vaccine
VDPV: Vaccine-derived poliovirus
(q)PCR: (Quantitative) polymerase chain reaction
Ct-value: Threshold cycle value
bp: Base pairs
UTR: Untranslated region
VP1: Virus protein 1
nt: Nucleotide
aa: Amino acid
